# The reasons to include the serology of human T-lymphotropic virus types 1 and 2 (HTLV-1 and HTLV-2) in the clinical follow-up of patients with viral hepatitis B and C in Brazil

**DOI:** 10.1371/journal.pntd.0008245

**Published:** 2020-05-26

**Authors:** Karoline Rodrigues Campos, Fabiana Aparecida Alves, Marcílio Figueiredo Lemos, Regina Célia Moreira, Rosa Maria Nascimento Marcusso, Adele Caterino-de-Araujo

**Affiliations:** 1 Laboratório de Pesquisa em HTLV, Centro de Imunologia, Instituto Adolfo Lutz, Coordenadoria de Controle de Doenças, Secretaria de Estado da Saúde, São Paulo, SP, Brasil; 2 Laboratório de Hepatites Virais, Centro de Virologia, Instituto Adolfo Lutz, Coordenadoria de Controle de Doenças, Secretaria de Estado da Saúde, São Paulo, SP, Brasil; 3 Ambulatório de HTLV, Serviço de Neurologia Clínica, Instituto de Infectologia Emilio Ribas (IIER), São Paulo, SP, Brasil; CSIR-Indian Institute of Chemical Biology, INDIA

## Abstract

**Background:**

The WHO established targets for 2030 to globally reduce new viral hepatitis B and C infections by 90% and deaths by 65% and recommends searching for coinfections that increase the progression of chronic liver infections towards cirrhosis and hepatocellular carcinoma.

**Aims and methodology:**

This study aimed to add information concerning the influence of human T-lymphotropic virus type 1 (HTLV-1) and type 2 (HTLV-2) infections in hepatitis B and C, since in Brazil, these human retroviruses are endemic but neglected. Serum samples from 1,910 patients with hepatitis B and 1,315 with hepatitis C from São Paulo, southeast Brazil, that were previously tested and grouped for HIV and HTLV-1/-2 coinfections were analyzed for hepatitis B virus (HBV) and hepatitis C virus (HCV) loads measurements and subsequent clearance using data from laboratory records.

**Key results:**

Briefly, the lowest HBV viral load (VL) was detected in HBV/HTLV-2 coinfected patients, regardless of whether they were infected with HIV (all comparisons *p*<0.05). In contrast, higher HCV VL was detected in HCV/HIV, HCV/HIV/HTLV-1/-2 coinfected patients (all *p*<0.05), and the lowest HCV VL was detected in HCV/HTLV-2 coinfected patients. Curiously, 61.1% of the patients with HBV/HTLV-2 coinfection had an undetectable HBV VL at the beginning of the study versus 21.4% in the patients with HBV/HTLV-1 coinfection. Although the percentages of undetectable HCV loads in HCV/HTLV-1 and HCV/HTLV-2 coinfected patients were quite similar, during follow-up, more HCV clearance was detected in patients with HCV/HTLV-2 coinfection [OR 2.65; 95% IC (1.17–5.99)].

**Major conclusions:**

HTLV-2 positively impacts HBV and HCV viral loads and HCV clearance, while HIV and/or HTLV-1 negatively impacts HCV viral load. Thus, the search for HTLV-1/-2 in viral hepatitis B and C infected patients has virological prognostic value, which is a strong reason to suggest including HTLV serology in the follow-up of patients.

## Author summary

The human T-lymphotropic viruses type 1 (HTLV-1) and type 2 (HTLV-2) are endemic and neglected in Brazil, and although these viruses were considered to have prognostic value when associated with HIV, e.g., HTLV-1 has been associated with fast progression and death to AIDS and more cases of lymphoma and neurological disorders, and HTLV-2 has been associated with slow progression to AIDS, their impact on hepatitis B virus (HBV) and hepatitis C virus (HCV) outcomes remain unknown. The present study analyzed the influence of HTLV-1 and HTLV-2 coinfection on HBV and HCV virological outcomes (viral load levels and virus clearance during follow-up) in serum samples from 1,910 patients with hepatitis B and 1,315 patients with hepatitis C from São Paulo, southeast Brazil. The results obtained confirmed the positive impact of HTLV-2 on HBV and HCV virological outcomes (lower HBV and HCV viral loads and increased HCV clearance when compared with HCV-monoinfected patients) and the opposite effect of HTLV-1; these findings are similar to the effects observed in HIV/AIDS patients. Taking into consideration these results and the differential impact of HTLV-1 and HTLV-2 in viral hepatitis B and C, we suggest including the serology of HTLV-1/2 in the follow-up of such patients in Brazil.

## Introduction

Viral hepatitis infections, mostly those caused by hepatitis B virus (HBV) and hepatitis C virus (HCV), are major global health problems. HBV and HCV can cause chronic infections, and a significant number of chronically infected individuals will develop cirrhosis and hepatocellular carcinoma [1–3]. In 2015, the World Health Organization (WHO) estimated 257 million people living with chronic hepatitis B and 887,000 deaths as a consequence of chronic hepatitis B. Nonetheless, a safe and effective vaccine that offers 98–100% protection against hepatitis B is available and could avert these deaths [1]. Concerning hepatitis C, the WHO estimated 71 million people living with chronic hepatitis C and approximately 400,000 deaths globally in 2016 as a consequence of cirrhosis and liver cancer. Currently, there is no effective vaccine against HCV infection, but antiviral medicines exist and can cure more than 95% of HCV-infected individuals, making access to diagnosis and treatment a good strategy for controlling this infection/disease [2]. In May 2016, the World Health Assembly adopted the first “Global Heath Sector Strategy on Viral Hepatitis 2016–2021: Towards Ending Viral Hepatitis”; the 2030 target is to reduce new viral infections by 90% and deaths by 65% [1,2]. The means to achieve these goals are as follows: (i) hepatitis B vaccination of infants with a 3-dose schedule as soon as possible after birth and of people in the high-risk group at any age; (ii) promote diagnosis using tests that could also differentiate acute from chronic infections and consequently assess the stage of liver disease; (iii) support universal treatment for viral hepatitis; (iv) prioritize treatment of people in advantageous stages of diseases; and (v) search for HBV and HCV in individuals at increased risk of viral infections, such as intravenous drug users (IDUs), prisoners, HIV-infected individuals, and others, to control transmission and liver disease progression. The Brazilian Ministry of Health (MH) signatory of this assembly since 2017 decided to first estimate the national number of viral hepatitis patients taking into account epidemiologic data, then establish an agenda regarding the number of patients to be tested, diagnosed and treated to achieve the WHO plan, and finally evaluate the costs to reach the WHO target [3,4]. The MH concluded that the WHO targets are feasible in Brazil with a scale-up of treatment and diagnosis over time, beginning in 2018 [4]. These target achievements are only possible because compulsory notification for viral hepatitis infections has been in place in Brazil since 1996 [5], and the Brazilian government has provided support for the universal treatment of patients [3,5]. Of note, at the present time, 233,027 cases of hepatitis B and 359,673 cases of hepatitis C were reported to the Brazilian MH from 1999 to 2018, with 34.9% (HBV) and 57.2% (HCV) from the southeast region of the country [6].

Unfortunately, human T-lymphotropic virus type 1 (HTLV-1), although endemic in Brazil, is responsible for at least two diseases of high morbidity and mortality [HTLV-1-associated myelopathy/tropical spastic paraparesis (HAM/TSP) and adult T cell leukemia/lymphoma (ATL), respectively] [7]; however, there is no obligatory notification for HTLV-1 infection and HTLV-1 is neglected in Brazil and worldwide [8]. The same mistreatment occurs with HTLV-2, which, although not associated with a specific disease, is related to neurological diseases similar to HAM/TSP and is endemic in Amerindians and IDUs from Brazil and elsewhere [9]. Curiously, when associated with HIV, HTLV-2 results in slow progression towards AIDS [10]. Conversely, HTLV-1, when associated with HIV, has been described to increase progression and death to AIDS [11,12]. Thus, the differential diagnosis of such retroviruses has prognostic value. Notably, coinfection by HIV and HBV as well as HIV and HCV have been collected by the Brazilian MH; coinfection rates of 5.2% and 7.8% (HIV/HBV), and 9.1% and 8.6% (HIV/HCV), were detected nationwide and in the southeast region, respectively [6].

Two years ago, we started studies on the prevalence and impact of HIV, HTLV-1 and HTLV-2 in patients with viral hepatitis B and C from São Paulo, southeast Brazil, and disclosed alarming results in hepatitis C, including high percentage of HCV/HTLV-1/-2 coinfection (5.3%) [13], and associations of HIV and/or HTLV-1 with high HCV viral load (VL) [14]. Unfortunately, in hepatitis B, although 1.3% of HBV/HTLV-1/2 coinfection was detected [13], the low number of patients had no statistical power for analysis. To confirm previous studies in hepatitis C and add information concerning hepatitis B, we conducted the present study.

## Methods

### Study population

The Instituto Adolfo Lutz (IAL), a Public Health Laboratory located in São Paulo City, is a reference laboratory for viral hepatitis, HIV, HTLV-1, and HTLV-2 infections. The IAL is responsible for the diagnosis and surveillance of these viruses and for assessing trends of infections and coinfections. Blood samples are sent to IAL from several Specialized Health Centers in the state of São Paulo, i.e., STI/AIDS and viral hepatitis centers and gastroenterology and/or hepatology services. A retrospective study was conducted using data obtained from cross-sectional studies previously described that utilized plasma samples collected from 1,910 patients analyzed for HBV VL at IAL during two periods: from June to November 2014 [13] and from June 2015 to June 2016 [15]. Serum samples were also collected from 1,315 patients analyzed for HCV VL during the same periods at IAL. The locations that sent these samples, as well as the sex, age, HIV and HTLV-1/2 infections data, were described elsewhere [13,15].

### Laboratory methods

All samples were first analyzed for HBV and HCV VL measurements at the IAL Viral Hepatitis Laboratory using Abbott Real-Time HBV and Abbott Real-Time HCV assays, with a lower limit of detection of 10 IU/mL and 12 IU/mL, respectively, and HCV genotyping using Abbott Real Time HCV Genotype II, (Abbott Molecular Inc., Illinois, USA). All assays were conducted according to the manufacturer’s instructions. Furthermore, the samples were tested for HIV-1, HTLV-1 and HTLV-2 coinfections at the IAL HTLV Research Laboratory using currently available assays for the analyses. Briefly, HIV-1 infection was evaluated using an immunochromatographic assay (Rapid Check HIV 1 + 2, Universidade Federal do Espírito Santo, ES, Brazil) and enzyme immunoassays (GS HIV-1/HIV-2 Plus O EIA, Bio-Rad Laboratories, USA, or Murex HIV Ag/Ab Combination, Diasorin, UK), as previously described [13,15]. Anti-HTLV-1/-2 antibodies were detected by enzyme immunoassays (EIA, HTLV-I/II, Gold ELISA, REM Ind. Com. Ltda, São Paulo, SP, Brazil, or EIA Murex HTLV-I/II, Diasorin, UK) and confirmed by Western Blot (WB) assay (HTLV BLOT 2.4, MP Biomedicals, Asia Pacific Pte Ltd.) and line immunoassay (INNO-LIA HTLV-I/II, Fujirebio, Europe N.V., Belgium). All assays were conducted according to the respective manufacturers’ instructions. The overall prevalence of HIV-1 detected by both studies was 8.4% and 11.5% in samples from patients with hepatitis B and C, respectively, and of HTLV-1/2, 1.7% in HBV and 4.6% in HCV patients, regardless of their HIV-1 status.

### Groups for viral load (VL) and clearance analysis and data collection

Six groups were categorized according to the results (positive or negative) for the following HIV-1 and HTLV-1/-2 serological assays. In hepatitis B: HBV mono-infection (n = 1,738); HBV/HIV-1 coinfection (n = 140), HBV/HTLV-1 coinfection (n = 7), HBV/HTLV-2 coinfection (n = 4), HBV/HIV/HTLV-1 coinfection (n = 7), and HBV/HIV/HTLV-2 coinfection (n = 14). Of note, because of the small sample size of the HBV/HTLV coinfection groups, primarily the HBV/HTLV-2 coinfection group (n = 4), four groups were categorized for the statistical analysis: HBV, HBV/HIV, HBV/HTLV-1 and HBV/HTLV-2 (the last two groups were irrespective of their HIV-1 status), although data of all groups were presented. In hepatitis C, initially six groups were categorized and statistically analyzed: HCV mono-infection (n = 1,127); HCV/HIV-1 coinfection (n = 127), HCV/HTLV-1 coinfection (n = 28), HCV/HTLV-2 coinfection (n = 9), HCV/HIV/HTLV-1 coinfection (n = 9), and HCV/HIV/HTLV-2 coinfection (n = 15). Subsequently, four groups were considered for clearance analysis: HCV, HCV/HIV, HCV/HTLV-1 and HCV/HTLV-2 (the last two groups were irrespective of their HIV-1 status).

HBV and HCV VL (log_10_ transformed), as well as the clearance results of HBV and HCV during two years of follow-up, were abstracted from laboratory records and analyzed according to the type of viral infection or coinfection.

### Statistical analyses

Differences in the numbers of males and females in each group were evaluated statistically using the Chi-square test. GraphPad Prism software version 5.03 (GraphPad, San Diego, CA, USA) was used for age and HBV and HCV VLs comparisons among three or more groups using Kruskal-Wallis analysis of variance (ANOVA), complemented with Dunn’s multiple-comparison test, and the Mann-Whitney U-test for comparing two groups. Results with a *p* value of <0.05 were considered statistically significant. Logistic regression univariate and multivariate analysis was used to identify the factors associated with HBV and HCV viral loads by calculating the odds ratios (ORs) and 95% confidence intervals (CIs). The analysis was performed using SPSS 21 (Statistical Package for the Social Sciences-21. Statistical Software. IBM, NY, USA).

### Ethical review

The study was approved by the IAL Ethics Committee for Research CTC#21I-2016 under Ministry of Health protocol number CAAE– 55837316.0.0000.0059. The data were analyzed anonymously.

## Results

The characteristics of the study populations according to group (mono-infection and coinfection) and the results of the HBV and HCV VL measurements detected in samples from patients with hepatitis B and hepatitis C during the first cross-sectional analysis are presented in Table 1 and Table 2, respectively.

**Table 1:** Characteristics of the study population and results of HBV viral load according to the type of infection and coinfection.

	Total	HBV	HBV/HIV	HBV/HTLV-1	HBV/HIV/HTLV-1	HBV/HTLV-1^a^	HBV/HTLV-2	HBV/HIV/HTLV-2	HBV/HTLV-2^a^	*p*
N (%)	1,910	1,738	140	7	7	14	4	14	18	
Male	1,013 (53.1)	877 (50.5)	116 (82.9)	3 (42.9)	4 (57.1)	7 (50.0)	2 (50.0)	11 (78.6)	13 (72.2)	<0.0001^d^
Female	896 (46.9)	860 (49.5)	24 (17.1)	4 (57.1)	3 (42.9)	7 (50.0)	2 (50.0)	3 (21.4)	5 (27.8)	
Age median^b^ (IQR)										
Male	47 (38–56)	47 (38–57)	47 (40–52)	60 (44–76)	47.5 (44.25–56)	50 (44–60)	55 (48–62)	47 (45–51)	47 (39–52)	
Female	45 (36–57)	45 (36–57)	45 (38.25–52.75)	66 (57.25–70.25)	62 (52–63)	63 (55–68)	46 (42–54)	44 (43–51)	44 (42.5–52.5)	
Total	46 (37–56)	46 (37–57)	46 (39–52)	64 (55–71)	52 (45–62)	59 (44–65)	51 (43.5–60)	47 (43.75–51)	47 (39–51.5)	
Viral Load median^c^ (IQR)	n = 1,349	n = 1,255	n = 76	n = 4	n = 7	n = 11^a^	n = 1	n = 6	n = 7^a^	
Male	2.67 (1.83–3.45)	2.68 (1.87–3.41)	2.59 (1.56–4.68)	2.45 (1.86–4.58)	3.79 (1.55–7.53)	2.50 (1.86–5.09)	-	1.48 (1.26–1.93)	1.48 (1.26–1.93)	
Female	2.60 (1.94–3.18)	2.61 (1.95–3.18)	2.36 (1.70–3.64)	2.37 (2.37–2.37)	1.95 (1.46–7.18)	2.16 (1.58–5.98)	1.74 (1.74–1.74)	2.19 (2.19–2.19)	1.96 (1.74–2.19)	
Total	2.63 (1.89–3.33)	2.64 (1.90–3.29)	2.48 (1.60–4.66)	2.41 (1.99–4.05)	2.50 (1.46–7.18)	2.45 (1.86–5.09)	1.74 (1.74–1.74)	1.61 (1.29–2.11)	1.61 (1.32–2.04)	

N, number; %, percentage

^a^, HBV/HTLV coinfected groups regardless of HIV status used for statistical analysis

^b^, age in years; IQR, interquartile range between 25% and 75%

^c^, viral load in log_10_. HBV viral load determination using the Abbott Real-Time HBV assay (Abbott Molecular Inc., IL, USA)

^d^, chi-square test.

**Table 2:** Characteristics of the study population and results of HCV viral load according to the type of infection and coinfection.

	Total	HCV	HCV/HIV	HCV/HTLV-1	HCV/HIV/HTLV-1	HCV/HTLV-2	HCV/HIV/HTLV-2	*p*
N (%)	1,315	1,127	127	28	9	9	15	
Male	733 (55.74)	581 (51.55)	109 (85.83)	17 (60.71)	6 (66.67)	7 (77.78)	13 (86.67)	<0.0001^c^
Female	582 (44.26)	546 (48.45)	18 (14.17)	11 (39.29)	3 (33.33)	2 (22.22)	2 (13.33)	
Age median^a^ (IQR)								
Male	50.0 (44–57)	51.0 (44–58)	46.0 (43–50)	54.0 (48–58)	44.0 (43–51.2)	46.0 (44–49)	47.0 (41–53.5)	
Female	53.0 (42–60)	53.0 (42–60)	47.0 (41.7–55)	51.0 (41–60)	51.0 (40–60)	46.5 (42–51)	59.5 (55–64)	
Total	51.0 (43–59)	52.0 (43–50)	46.0 (43–50)	53.5 (47–58)	44.0 (43–51.5)	46.0 (43–50)	49.0 (42–55)	
Viral Load median^b^ (IQR)	n = 925	n = 787	n = 90	n = 25	n = 5	n = 8	n = 10	
Male	5.88 (5.20–6.32)	5.77 (5.13–6.28)	6.16 (5.75–6.52)	5.86 (5.74–6.23)	6.82 (6.73–6.89)	5.25 (3.69–6.26)	6.48 (6.35–6.89)	
Female	5.60 (4.91–6.12)	5.56 (4.90–6.11)	5.81 (5.43–6.25)	5.59 (5.08–6.20)	6.52 (6.17–6.87)	4.81 (4.02–5.60)	5.71 (5.71–5.71)	
Total	5.75 (5.07–6.26)	5.66 (5.02–6.20)	6.10 (5.72–6.46)	5.84 (5.35–6.20)	6.82 (6.45–6.88)	5.25 (3.99–5.91)	6.48 (6.17–6.87)	

N, number; %, percentage

^a^, age in years; IQR, interquartile range between 25% and 75%

^b^, viral load in log_10_. HCV viral load determination using the Abbott Real-Time HCV assay (Abbott Molecular Inc., IL, USA)

^c^, chi-square test.

**Fig 1:**
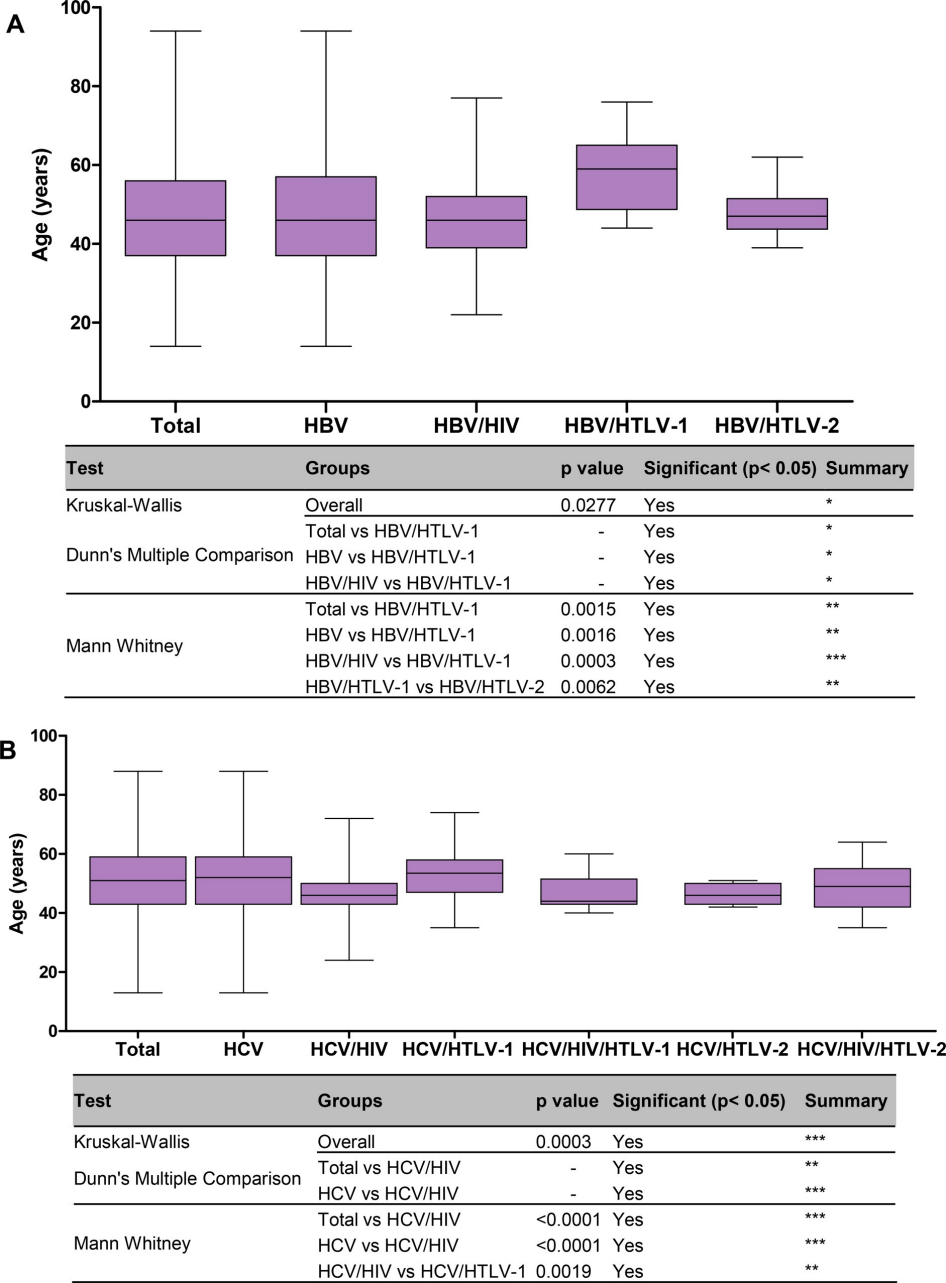
**Median age of patients with hepatitis B (A) and hepatitis C (B) according to the type of viral infection and coinfection.** The Kruskall-Wallis test was used for statistical analysis (three groups or more), complemented with Dunn’s Multiple Comparison test, and the Mann-Whitney U-test (two groups). *p*-values depicted as asterisks correspond to:*, *p*<0.05; **, *p*≤0.01; *** *p*≤0.001.

Briefly, in patients with hepatitis B, more men were coinfected with HIV and HIV/HTLV-2 when compared to the other groups (all *p*<0.0001). The patients with the oldest median age belonged to the HBV/HTLV-1 coinfection group (median age of 64 years) (Table 1), with statistically significant differences when compared with HBV mono-infection (*p*<0.05) and HBV/HIV coinfection groups of patients (*p*<0.05, using Kruskal-Wallis and Dunn’s multiple comparison tests). Results with statistical significance detected by Mann-Whitney U-test were depicted in Fig 1A.

**Fig 2:**
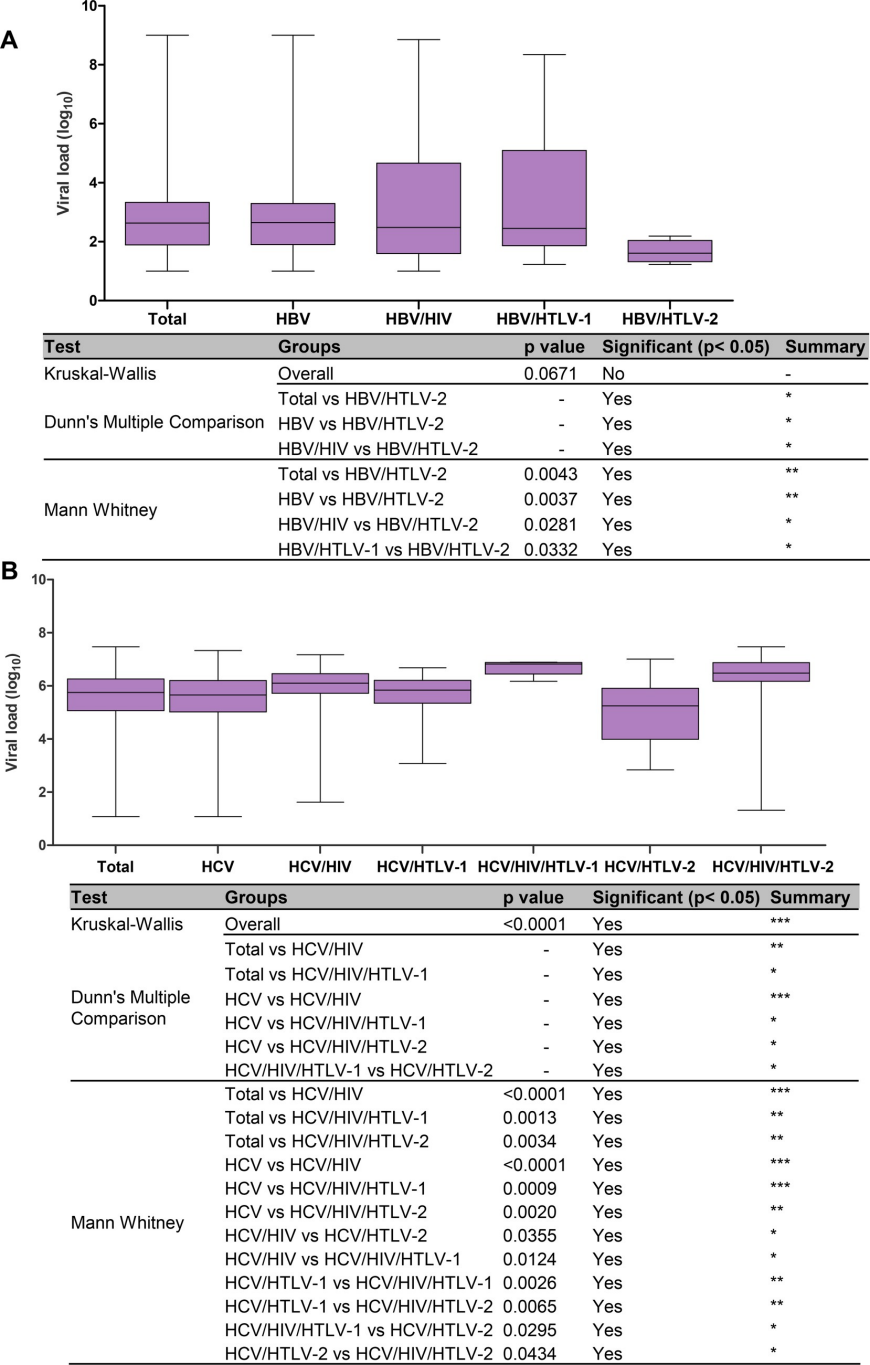
**HBV (A) and HCV (B) viral load results according to the type of viral infection or coinfection.** The Kruskall-Wallis test was used for statistical analysis (three groups or more), complemented with Dunn’s Multiple Comparison test, and the Mann-Whitney U-test (two groups). *p*-values depicted as asterisks correspond to:*, *p*<0.05; **, *p*≤0.01; *** *p*≤0.001.

In relation to HBV VL, a minor HBV VL was detected in HBV/HTLV-2 coinfected patients irrespective of their HIV status (1.61 log_10_ IU/mL), which was 1.03 log_10_ lower than the VL value of the HBV mono-infected group (2.64 log_10_ IU/mL) (Table 1, in evidence). Significant differences were detected among groups when HBV/HTLV-2 VL levels were compared with the values obtained in the HBV mono-infection and HBV/HIV coinfection groups (both *p*<0.05 by Kruskal-Wallis and Dunn’s multiple comparison tests), and the results with significance obtained by Mann-Whitney U-test were presented in Fig 2A. Briefly, they confirmed the benefit of HTLV-2 on HBV viral load (HBV VL lower levels).

In relation to hepatitis C, the majority of HIV and/or HTLV coinfected patients were men, and this differed significantly in relation to the HCV mono-infected group (Chi-square *p*<0.0001) (Table 2). Concerning age, although not as high as in the HBV/HTLV-1 study group (median age 64 years), the oldest median age was also detected in HCV/HTLV-1 coinfected patients (53.5 years). But, statistically significant differences by Kruskal-Wallis test was detected only when comparing the ages of HCV mono-infected with HCV/HIV coinfected patients (*p*≤0.001, Fig 1B). By Mann-Whitney U-test significant differences in age were detected when compared HCV mono-infected with HCV/HIV coinfected patients (*p*<0.0001), and HCV/HIV with HCV/HTLV-1 coinfected patients (*p* = 0.0019), Table 2, and Fig 1B. Regarding HCV VL measurements, overall, the median value was 5.75 log_10_ IU/mL, and the VL was 0.28 log_10_ higher in men than in women. Higher HCV VL values were detected in HCV/HIV, HCV/HIV/HTLV-1, and HCV/HIV/HTLV-2 coinfection groups, with the highest values observed in the HCV/HIV/HTLV-1 coinfected patients (6.82 log_10_ IU/mL) (Table 2, Fig 2B). In contrast, the lowest HCV VL value was detected in HCV/HTLV-2 coinfected patients (5.25 log_10_ IU/mL) (Table 2, Fig 2B). The statistically significant differences among groups using both non parametric tests (Kruskal-Wallis and Mann-Whitney U-test) are depicted in Fig 2B. Briefly, they confirmed association of HIV and HTLV-1 with high HCV VL, and HTLV-2 with the low HCV VL.

When we analyzed the number of samples that resulted in VLs that were detectable or under the detection limit of the applied assay (VL undetectable) at the first evaluation, a larger percentages of HBV undetectable VL samples were observed in HBV/HTLV-2 individuals (61.1%), contrasting with the lowest value observed in HBV/HTLV-1 coinfected individuals (21.4%). Using univariate logistic regression analysis the HBV/HIV and the HBV/HTLV-2 coinfected individuals presented more odds to undetectable HBV VL measurements than the HBV mono-infected counterpart [OR = 2.19 (95% CI, 1.54–3.10) and OR = 4.08 (95% CI, 1.57–10.60), respectively], Table 3. Indeed, during follow-up, the same percentage of individuals continued with undetectable HBV viral loads in the HBV/HTLV-2 group (61.1%) and increased somewhat in HBV/HTLV-1 patients (35.7%) (data obtained from laboratory records).

**Table 3:** Factors associated with undetectable HBV viral load in HBV infection and coinfection at the beginning of study.

			Univariate analysis				
				HBV^a^	HBV/HIV	HBV/HTLV-1	HBV/HTLV-2
				n = 1,738	n = 140	n = 14	n = 18
**Male**		OR			4.74	0.98	2.55
		[95%CI]			[3.02–7.43]	[0.34–2.81]	[0.91–7.18]
		*p* Wald			≤0.001	0.981	0.077
**Age ≤ 50 years**		OR			1.22	0.25	1.25
		[95%CI]			[0.85–1.76]	[0.08–0.80]	[0.47–3.34]
		*p* Wald			0.286	0.019	0.662
**Undetectable HBV viral load**		OR			2.19	0.71	4.08
		[95%CI]			[1.54–3.10]	[0.20–2.55]	[1.57–10.60]
		*p* Wald			≤0.001	0.589	0.004

n, number; OR, odds ratio; 95% CI, 95% confidence interval

^a^ reference group.

When the same analysis was conducted in HCV patients, no difference in the percentage of undetectable VL levels was observed between the HCV/HTLV-1 patients (18.9%) and HCV/HTLV-2 patients (25.0%). However, in contrast, during follow-up, 50.0% of the HCV/HTLV-2 coinfected patients converted to an undetectable HCV VL, contrasting with 35.1% in HCV/HTLV-1 coinfected patients. The univariate and multivariate regression analysis confirmed the benefit of HTLV-2 infection on HCV clearance [OR = 2.56; 95% CI (1.14–5.75) and OR = 2.65 (1.17–5.99), respectively] when compared HCV/HTLV-2 coinfected patients with HCV mono-infected counterpart (Table 4).

**Table 4:** Factors associated with HCV clearance in HCV infection and coinfection.

			Univariate analysis					Multivariate analysis			
				HCV^a^	HCV/HIV	HCV/HTLV-1	HCV/HTLV-2		HCV^a^	HCV/HIV	HCV/HTLV-2
				n = 1,127	n = 127	n = 37	n = 24		n = 1,127	n = 127	n = 24
**Male**		OR			5.69	1.54	4.70	OR		5.29	4.66
		[95%CI]			[3.41–9.50]	[0.79–3.03]	[1.60–13.83]	[95% CI]		[3.16–8.86]	[1.58–13.79]
		*p* Wald			≤0.001	0.207	0.005	*p* Wald		≤0.001	0.005
**Age ≤ 50 years**		OR			3.70	0.73	2.40	OR		3.50	2.16
		[95%CI]			[2.43–5.64]	[0.37–1.43]	[1.02–5.63]	[95% CI]		[2.28–5.37]	[0.91–5.13]
		*p* Wald			≤0.001	0.356	0.046	*p* Wald		≤0.001	0.080
**HCV Clearance**		OR			0.63	1.38	2.56	OR		0.64	2.65
		[95%CI]			[0.40–0.99]	[0.70–2.75]	[1.14–5.75]	[95% CI]		[0.40–1.03]	[1.17–5.99]
		*p* Wald			0.044	0.354	0.023	*p* Wald		0.064	0.020

n, number; OR, odds ratio; 95% CI, 95% confidence interval

^a^ reference group.

Of note, using univariate analysis, male sex and the age of ≤50 years were factors associated with HBV/HIV and HBV/HTLV-2 coinfections (Table 3). The same was observed in hepatitis C using univariate analysis, both, male sex and age of ≤50 years were factors associated with HCV/HIV and HCV/HTLV-2 coinfections (Table 4).

Notably, according to laboratory records, the most prevalent HCV genotypes identified in samples of individuals with hepatitis C were genotype 1a (42.8%), followed by genotypes 1b (28.4%), 3 (14.1%), and 3a (6.0%). No considerable differences in genotypes according to groups (HCV mono-infection or coinfection) were detected (data obtained from laboratory records).

## Discussion

To reach the 2030 WHO goal to reduce viral hepatitis B and C infections by 90% and death by 65%, a search for coinfections (mostly HIV) that could increase the progression of liver chronic infections towards cirrhosis and hepatocellular carcinoma has been recommended, but HIV is not the only retrovirus associated with liver disease progression in viral hepatitis. Some studies from Japan and elsewhere have reported notably worse virological and clinical outcomes in hepatitis C patients coinfected with HTLV-1, i.e., higher HCV viremia, lower HCV sustained virological response to α-interferon treatment, increased risk of cirrhosis and greater liver cancer mortality when compared to their counterparts with HCV infection alone [15–18]. No effort to search for HTLV-1/2, which are endemic in most areas, has been made by the WHO, even though the WHO currently encourages a healthy sexual life and the availability of testing for other blood borne and sexually transmitted viruses, such as hepatitis B and C [1,2]. Of note, a letter sent to the WHO by Fabiola Martin, Yutaka Tagaya, and Robert Gallo, signed by more than 50 researchers and organizations with concern about HTLV explains why now is the time to eradicate HTLV-1 [8].

Although there is a lack of information relating to the impact of HTLV-1/2 on HBV disease progression and clearance, the present study discloses important results. In hepatitis B, we first confirmed more HIV and HIV/HTLV-2 coinfection among men, which could be explained, in part, by men having a greater number of sexual partners during their lifetime; additional, there are more IDUs among males. In fact, corroborating this hypothesis, HTLV-2 has been detected and associated with IDU mostly in male HIV-infected patients in São Paulo (southeast Brazil) and in Londrina (southern Brazil) [19,20]. Additionally, in Salvador (northeast Brazil), HBV predominates among males infected with HIV and coinfected with HIV/HTLV-1, and the sexual route was assigned as the major risk factor for acquiring such retroviruses in this geographic region [21]. In relation to age, patients coinfected by HBV/HTLV-1/2 were older, and this could be due in part to the institution of mandatory serology for HBV and HIV in blood banks in Brazil in 1988 and for HTLV-1/2 and HCV in 1993 [22,23]; additionally, IDU was more common in Brazil in the 1980s and 1990s [24].

Concerning the impact of HTLV-1 and HTLV-2 on HBV virological outcomes, in HBV/HTLV-1 coinfected patients from Salvador, a higher prevalence of HBsAg (evidence of persistent HBV infection or reduced spontaneous clearance of HBV) was detected when compared with HBV mono-infected patients [21]. Another recent study conducted in individuals from the Northern Territory of Australia, which is endemic for HBV and HTLV-1 infections, disclosed a higher prevalence of HBsAg and anti-HBc seropositivity and a lesser degree of HBV clearance in HTLV-1-infected individuals than in HTLV-1 non-infected individuals [25]. These studies corroborate the results presented herein and confirm a negative impact of HTLV-1 on HBV virological outcome, which could influence liver disease progression.

On the other hand, the present study confirms, for the first time, the benefit of HTLV-2 infection on HBV clearance. In fact, at the beginning of the study, more patients coinfected with HBV/HTLV-2 had spontaneous clearance of HBV compared with the HBV/HTLV-1 coinfected patients, regardless of their HIV status. The mechanism by which HTLV-2 affects HBV VL and clearance is unknown and deserves further study. However, we could speculate that the increase in the number of cytotoxic CD8 T lymphocyte responses in HTLV-2 infection could control HBV replication, as reported in HIV and in HCV replication control in studies conducted elsewhere [10,26].

In relation to hepatitis C, as expected among retrovirus coinfected patients, males predominated. This could be explained by the same risk factors described for HBV and mostly by the IDU, since HCV has been strongly associated with this route of virus transmission [6,19–21]. In relation to age, although HCV/HTLV-1/2 coinfected patients disclosed older ages, significant differences were detected only between HCV/HIV coinfected patients (who had the youngest median age) in relation to the ages of HCV and HCV/HTLV-1 groups. This result raises the possibility of more recent HIV infection, probably by the sexual route, as documented by the Brazilian MH, which showed an increase in the number of HIV-infected individuals in the second and third decades of life, mostly by homosexual practices [27], although the parenteral route could not be excluded in this group of patients.

Concerning HCV virological outcomes in mono-infected and coinfected individuals, the results obtained herein confirmed the negative impact of HIV and/or HTLV-1 on HCV VL and clearance, as well as the higher VL levels in males than in females, as previously described [14], and add information on HTLV-2 coinfection. Although some studies in Salvador, Bahia, have pointed to an advantage among HCV/HTLV-1 coinfected individuals in promoting HCV clearance, probably by cytokine production [28,29], other studies conducted around the world, including one from São Paulo and the present study, reported the opposite effect [14,16,17]. Differences in the genetic background of patients, diversity of HTLV-1, HTLV-2 and HCV strains, and the number and characteristics of the recruited study populations could account for the discordant results.

In the present study, we were able to analyze and compare HCV VL and clearance in 787 HCV-infected, 90 HCV/HIV coinfected, and 48 HCV/HTLV-1/2 coinfected individuals from São Paulo (a state with a mixed-race population where HTLV-1aA, HTLV-2c subtypes, and HCV genotype 1 predominate). We confirmed higher HCV VL and viremia and lower HCV clearance during follow-up among HCV/HTLV-1 coinfected patients than in HCV mono-infected and HCV/HTLV-2 coinfected patients.

Of interest, although lowest HCV VL values were detected in HCV/HTLV-2 coinfected patients, when the patients were also infected by HIV, the HCV VL increased, showing that HIV takes advantage of HTLV-2 coinfection in patients with hepatitis C, probably during the acute or primary HIV infection. However, after treatment (Table 4) and during follow-up, HCV/HTLV-2 coinfected individuals showed more HCV clearance, emphasizing the HTLV-2 protective role in HCV virological outcomes and consequent disease progression. Notably, the Brazilian MH has provided free and universal access to interferon-free direct-acting antivirals (DAAs) for hepatitis C treatment in the country since 2014 [30]. The mechanism by which HTLV-2 acts in hepatitis C is unknown, but again, high CD8 T cell counts associated with HCV replication control have been described in patients coinfected by HTLV-2 and in HIV-1 controllers, both with a history of injection drug use, in Spain [26], corroborating the present data. In addition, differences between the fitness of HIV and HTLV-2 could account for the initial result obtained. Since HTLV-2 has been pointed as a protective factor for HIV/AIDS progression, probably by cytokines production that blocks the CCR5 coreceptor of HIV [10,31], we could hypothesis that at the beginning of HIV and HTLV-2 coinfection, and/or before antiretroviral therapy (ART), the HIV takes advantage of the HTLV-2 (high viral load levels). But during chronic HIV-1 and HTLV-2 coinfection, and in patients on ART, the HTLV-2 could takes advantage on HIV, and could control the HCV viral load. In fact, corroborating this hypothesis during follow-up of two years, more HCV clearance was detected in HCV/HTLV-2 coinfected individuals regardless of their HIV status.

Other factors, such as the genetic background of individuals, the subtype of HTLV-2 strain, the Tax 2 protein, and the antisense protein APH-2, among others, could also be implicated in the benefit of HTLV-2 in hepatitis C, as occurs in HIV/AIDS [10,14,16,26].

Additionally, the results described herein concerning the HCV genotypes detected among patients corroborate the genotypes that are prevalent in Brazil [5], and no association was detected between one specific genotype and group of mono-infected or coinfected patients.

Remarkably, some Brazilian public health policies concerning HTLV were implemented in recent years in Brazil due to the pressure of clinicians, researchers, HTLV-infected individuals and data from the literature. All of these policies stressed the importance of the correct diagnosis of HTLV-1 and HTLV-2 for monitoring and adequately treating such individuals. They included the recommendation of HTLV-1/2 serology at the first clinical visit for HIV-infected individuals of regions where HTLV-1 is endemic [27]; the use of confirmatory (Western Blot or PCR) tests for HTLV-1 in the Brazilian public health care system in cases suspected of ATLL [32]; and the use of zidovudine to treat adult leukemia/lymphoma associated with HTLV-1 [33]. On the other hand, neither serology nor treatment were considered by the Brazilian MH for cases suspected of HAM/TSP, although Brazil accounts for the largest number of cases in the world. Recently, (April 2019) HAM/TSP received the attention of the WHO; it was given a distinct International Classification of Disease code (code: 8A45.00) (http://id.who.int/icd/entity/1043229589).

Finally, the major limitations of the present study was the low number of HTLV-1 and HTLV-2 coinfected patients in each HBV and HCV subgroup analyzed, but we have to consider that the samples came from the Viral Hepatitis Laboratory and not from HTLV out-patients clinics. Thus, the numbers of HBV/HTLV-1/2 and HCV/HTLV-1/2 coinfected individuals although low, are in accordance with the HTLV-1/2 numbers detected in high-risk populations, such as in HIV/AIDS patients [19,34], and in endemic populations in Brazil [35–36]. Another limitation of this study was the fact that we unknown the HIV viral load levels in HIV and/or HIV/HTLV-1/2 coinfected individuals, but using the regression univariate and multivariate analysis we tried to circumvent this lack of information. Even though the low samples size seems to have no power to ascertain the negative impact of HTLV-1 and the positive impact of HTLV-2 on hepatitis B and C virological outcomes, the results obtained allow us to suggest introducing the serology of HTLV in the follow-up of patients with hepatitis B and C in Brazil, making visible the HTLV-1 and HTLV-2 invisible infections. More studies have to be conducted in Brazil and elsewhere to confirm the present results.

In conclusion, taking into consideration the results obtained and the different impact of HTLV-1 and HTLV-2 on HBV and HCV loads and clearance, together with the WHO plan for success in Brazil, we suggest including HTLV-1/2 serology in the battery of tests used in the follow-up of patients with viral hepatitis B and C in this country.

## Supporting information

S1 Checklist STROBE statement. (DOC)
