# Supplementary material for: The reasons to include the serology of human T-lymphotropic virus types 1 and 2 (HTLV-1 and HTLV-2) in the clinical follow-up of patients with viral hepatitis B and C in Brazil
Source: PLoS Negl Trop Dis. 2020 May 26;14(5):e0008245. doi: 10.1371/journal.pntd.0008245 (PMC7274452; doi:10.1371/journal.pntd.0008245)
Supplement: S1 Checklist — (DOC) [file pntd.0008245.s001.doc]

STROBE Statement—Checklist of items that should be included in reports of ***cross-sectional studies***

|  | Item No | Recommendation |
| --- | --- | --- |
| **Title and abstract** | 1 | (*a*) Abstract, paragraph 2 |
|  |  | (*b*) Abstract, paragraphs 2 and 3 |
| Introduction | | |
| Background/rationale | 2 | Introduction, paragraphs 1 and 2 |
| Objectives | 3 | Introduction, paragraph 3 |
| Methods | | |
| Study design | 4 | Study population, paragraph 1 |
| Setting | 5 | Study population, paragraph 1 |
| Participants | 6 | Laboratory methods, Groups for viral load (VL) and clearance analysis and data collection, paragraphs 2 and 3 |
| Variables | 7 | Laboratory methods, Groups for viral load (VL) and clearance analysis and data collection, paragraphs 2 and 3 |
| Data sources/ measurement | 8* | Laboratory methods, paragraph 2 |
| Bias | 9 | Groups for viral load (VL) and clearance analysis and data collection, paragraph 3 |
| Study size | 10 | Study population, paragraph 1 |
| Quantitative variables | 11 | Groups for viral load (VL) and clearance analysis and data collection, paragraph 3 |
| Statistical methods | 12 | (*a*) Statistical analysis, paragraph 4 |
|  |  | (*b*) Statistical analysis, paragraph 4 |
|  |  | (*c*) Statistical analysis, paragraph 4 |
|  |  | (*d*) Statistical analysis, paragraph 4 |
|  |  | (*e*) not applicable |
| Results | | |
| Participants | 13* | (a) Results, paragraph 1, Tables 1 and 2 |
|  |  | (b) not applicable |
|  |  | (c) not applicable |
| Descriptive data | 14* | (a) Results, Tables 1 and 2 |
|  |  | (b) not applicable |
| Outcome data | 15* | Results, paragraphs 3 and 4, Tables 1 and 2, and Figures 1 and 2 |
| Main results | 16 | (*a*) Results, paragraphs 2, 3 and 4, Figures 1 and 2 |
|  |  | (*b*) Results, paragraphs 2, 3 and 4, Tables 1 and 2, Figures 1 and 2 |
|  |  | (*c*) Results, paragraphs 5, 6 and 6, Tables 3 and 4 |
| Other analyses | 17 | Results, paragraph 8 |
| Discussion | | |
| Key results | 18 | Discussion, paragraphs 3, 4, 5, 6, and 7 |
| Limitations | 19 | Discussion, paragraphs 12 |
| Interpretation | 20 | Discussion, paragraphs 8 and 9 |
| Generalisability | 21 | Discussion, paragraphs 1, 2, 8, 10, 11 and 13 |
| Other information | | |
| Funding | 22 | Fundação de Amparo a Pesquisa do Estado de São Paulo (FAPESP), grants N° 2016/03654-0 |

*Give information separately for exposed and unexposed groups.

**Note:** An Explanation and Elaboration article discusses each checklist item and gives methodological background and published examples of transparent reporting. The STROBE checklist is best used in conjunction with this article (freely available on the Web sites of PLoS Medicine at http://www.plosmedicine.org/, Annals of Internal Medicine at http://www.annals.org/, and Epidemiology at http://www.epidem.com/). Information on the STROBE Initiative is available at www.strobe-statement.org.
